# TRANSLATIONAL ADVISORY BOARDS IN RESEARCH: EXPERIENCES FROM A PRAGMATIC RESEARCH TRIAL

**DOI:** 10.1093/geroni/igz038.505

**Published:** 2019-11-08

**Authors:** Keith A Anderson, Holly Dabelko-Schoeny, Laura N Gitlin, Joseph E Gaugler, Sokha Koeuth, Katherine A Marx

**Affiliations:** 1 University of Montana, Missoula, Montana, United States; 2 The Ohio State University, Columbus, Ohio, United States; 3 Drexel University, Philadephia, Pennsylvania, United States; 4 University of Minnesota - School of Public Health, Division of Health Policy and Management, Minneapolis, Minnesota, United States; 5 Johns Hopkins University, Baltimore, Maryland, United States

## Abstract

Translational Advisory Boards (TABs) are select groups of researchers, practitioners, and, in some cases, service recipients (e.g., patients, caregivers) that convene regularly to advise researchers on ongoing studies. TABs provide direction and support, knowledge and insight, alternative points of view, and suggestions for overcoming obstacles and improving research functions. TABs are especially valuable in applied research in which “real world” conditions create challenges ranging from the anticipated (e.g., participant drop out) to the unanticipated (e.g., government shutdown). In this presentation, the researchers evaluate one TAB involved with an ongoing pragmatic research trial of Adult Day Services Plus (ADS Plus), an intervention for family caregivers to persons with dementia. Including individuals with diverse points of view was critical in the composition of the TAB. The TAB consisted of four seasoned researchers, four ADS industry professionals, and six program directors from the ADS Plus treatment and control groups. Creating meetings that were productive was imperative. TAB sessions were held bi-annually and had highly structured agendas soliciting guidance on specific issues. Qualitative analysis of the TAB sessions revealed three themes: assistance with recruitment; fidelity of the intervention; and sustainability of the intervention. This guidance helped the researchers to shift directions in recruitment and to consider further refinement of the intervention to increase sustainability. In looking at costs, the annual research personnel, TAB personnel, and overhead expenses were estimated at $2,500-$3,000 and $4,000-$5,500, respectively. In conclusion, TABs can be an effective tool to support research and should be considered for use in large-scale projects.

